# Sphincter damage during fistulotomy for perianal fistulae and its relationship with faecal incontinence

**DOI:** 10.1007/s00423-021-02307-5

**Published:** 2021-09-01

**Authors:** Stephanie García-Botello, Marina Garcés-Albir, Alejandro Espi-Macías, David Moro-Valdezate, Vicente Pla-Martí, Jose Martín-Arevalo, Joaquín Ortega-Serrano

**Affiliations:** 1 Colorectal Surgery Unit, Department of General and Digestive Surgery, Biomedical Research Institute INCLIVA, Hospital Clínico Universitario, Avd. Blasco Ibañez, 17, 46010 Valencia, Spain; 2 Department of Surgery, Universidad de Valencia, Valencia, Spain

**Keywords:** Fistulotomy, Anal ultrasound, Faecal incontinence, Perianal fistulae, Fistula, Incontinence, Fistulotomy

## Abstract

**Background:**

The length of sphincter which can be divided during fistulotomy for perianal fistula is unclear. The aim was to quantify sphincter damage during fistulotomy and determine the relationship between such damage with symptoms and severity of faecal incontinence and long-term quality of life (QOL).

**Methods:**

A prospective cohort study was performed over a 2-year period. Patients with intersphincteric and mid to low transsphincteric perianal fistulas without risk factors for faecal incontinence were scheduled for fistulotomy. All patients underwent 3D endoanal ultrasound (3D-EAUS) pre-operatively and 8 weeks postoperatively. Measurements were taken of pre- and postoperative anal sphincter involvement and division. Anal continence was assessed using the Jorge-Wexner scale and QOL scores pre, 6 and 12 months postoperatively.

**Results:**

Forty-nine patients were selected. A strong correlation between pre- and postoperative measurements was found *p* < 0.001. A median length of 41% of the external anal sphincter and 32% of the internal anal sphincter was divided during fistulotomy. Significant differences in mild symptoms of anal continence were found with increasing length of external anal sphincter division. But there was no significant deterioration in continence, soiling, or quality of life scores at the 1-year follow-up. Division of over two-thirds of the external anal sphincter was associated with the highest incontinence rates.

**Conclusions:**

3D-EAUS is a valuable tool for quantifying the extent of sphincter involvement pre- and postoperatively. Post-fistulotomy faecal incontinence is mild and increases with increasing length of sphincter division but does not affect long-term quality of life.

## Introduction

Fistulotomy is frequently used for the treatment of simple perianal fistula; however, the fistula height and sphincter length which can be divided during fistulotomy remain controversial. This technique is generally reserved for low, uncomplicated fistulae, albeit the exact amount of sphincter which defines a high or low fistula has not been established. Murad-Regadas et al. reported tracts crossing the external anal sphincter (EAS) at the same height or above the internal opening (IO) involved over 50% of the EAS and defined them as high fistulous tracts [1]. Conversely, other authors such as van Koperen [2] defined low fistulas as those which crossed the inferior third of the EAS. The classification of fistulas with respect to where they cross the EAS is purely arbitrary and varies between surgeons. In the majority of cases, it is related to the treatment considered most appropriate to obtain the highest cure rate and lowest postoperative sequelae. There is an overall 45% rate of postoperative faecal incontinence (FI). Risk of FI is associated with females, the surgical technique, a previous history of fistula surgery and high fistulae [3]. There is currently no firm evidence correlating rates of postoperative FI with length of sphincter division, and this remains a grey area of colorectal surgery [4, 5].

Three-dimensional endoanal ultrasound (3D-EAUS) has been shown to provide reliable, quantitative measurements of the anal sphincters both pre- and post-fistulotomy [4, 6–8] and is increasingly used by surgeons to help choose the best treatment options for patients with perianal fistulae [1, 5].

The aim of this study was to quantify sphincter damage during fistulotomy and determine the relationship between such damage with symptoms and severity of FI and quality of life (QOL).

## Materials and methods

A prospective cohort, single-centre, consecutive study was performed over a 2-year period including patients from a highly specialized colorectal unit in a tertiary referral centre. The study protocol was approved by the hospital ethics committee. All patients signed an informed consent form prior to inclusion in the study and were followed up for 1 year. Patients diagnosed with a simple cryptoglandular perianal fistula scheduled for fistulotomy were selected. Patients were excluded if operated in another centre, diagnosed with inflammatory bowel disease, had risk factors for FI, supra- or extrasphincteric fistulas or were currently undergoing treatment with other non-surgical techniques or medication.

A simple fistula was defined as an intersphincteric fistula or transsphincteric fistula which involved less than 66% of the total length of the external anal sphincter (EAS) in patients without risk factors for FI [5]. Risk factors for FI included female patients with anterior transsphincteric fistulas, obstetric injuries to the anal sphincters, inflammatory bowel disease and patients with prior anal surgery and major FI according to the Jorge-Wexner score [9]. Any extent of impairment in FI even in its mildest form was taken into account. Minor incontinence was defined as involuntary passage of gas or liquid and major incontinence as the involuntary loss of stools. Soiling was defined as the involuntary loss of small quantities of stool after defecation in patients who were otherwise continent [10]. Recurrence was defined as postoperative persistence or reappearance of symptoms. Pre- and postoperative 3D-EAUS measurements were taken together with assessment of FI and QOL.

### Study protocol

#### Preoperative assessment

A full medical history was taken with particular attention to previous anal surgeries, obstetric history and risk factors for FI measured by the Jorge-Wexner score [9] and QOL as previously mentioned. During the same visit, the presence of an IO, external opening (EO) and location of the fistula were documented by digital rectal examination.

#### 3D-EAUS

**Fig. 1:**
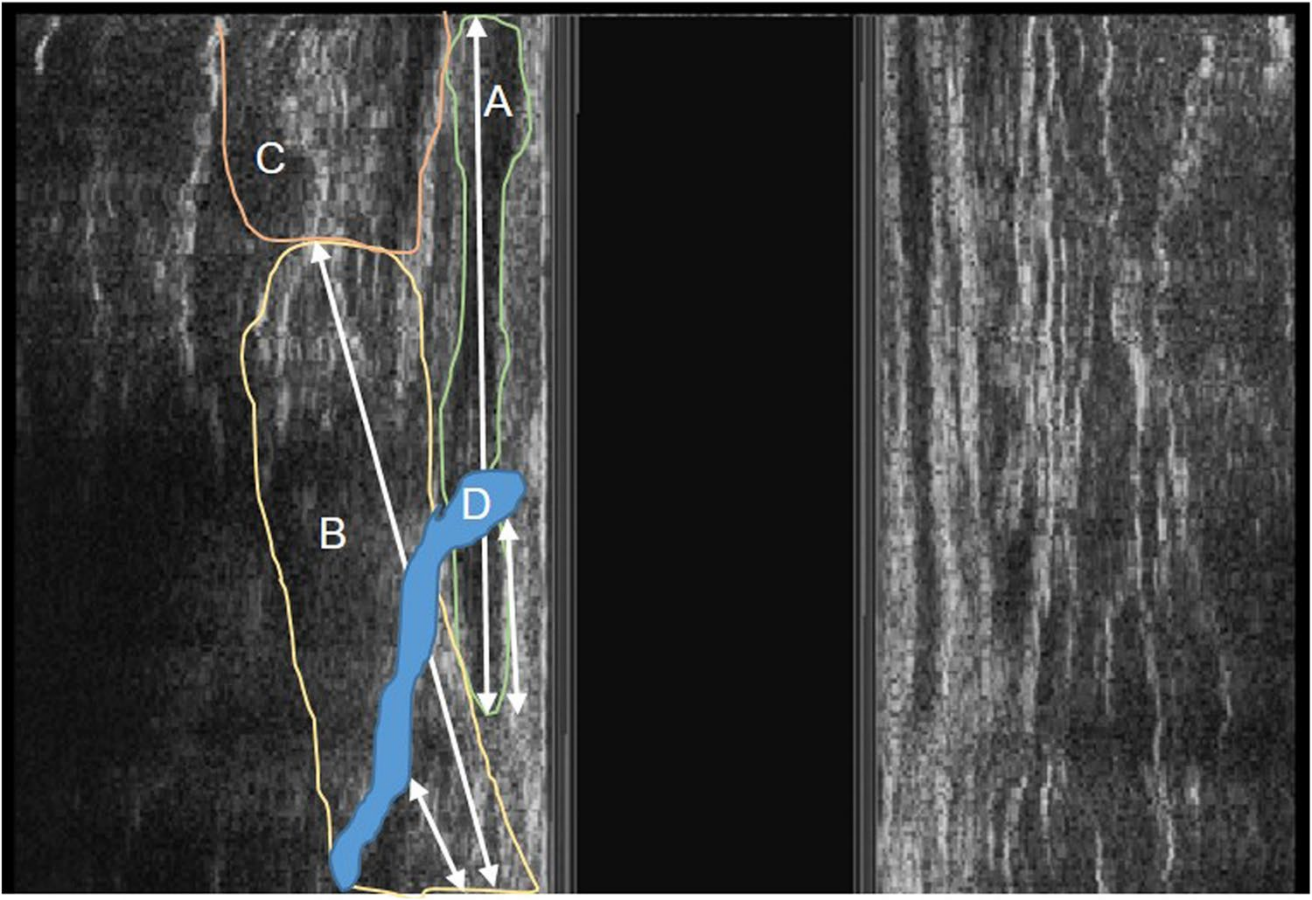
Endoanal ultrasound image showing a coronal section of a right lateral transphincteric fistula and arrows indicating total length and involved sphincter of both the internal and external anal sphincters. **A** internal anal sphincter, **B** external anal sphincter, **C** puborectalis muscle, **D** fistula tract

**Fig. 2:**
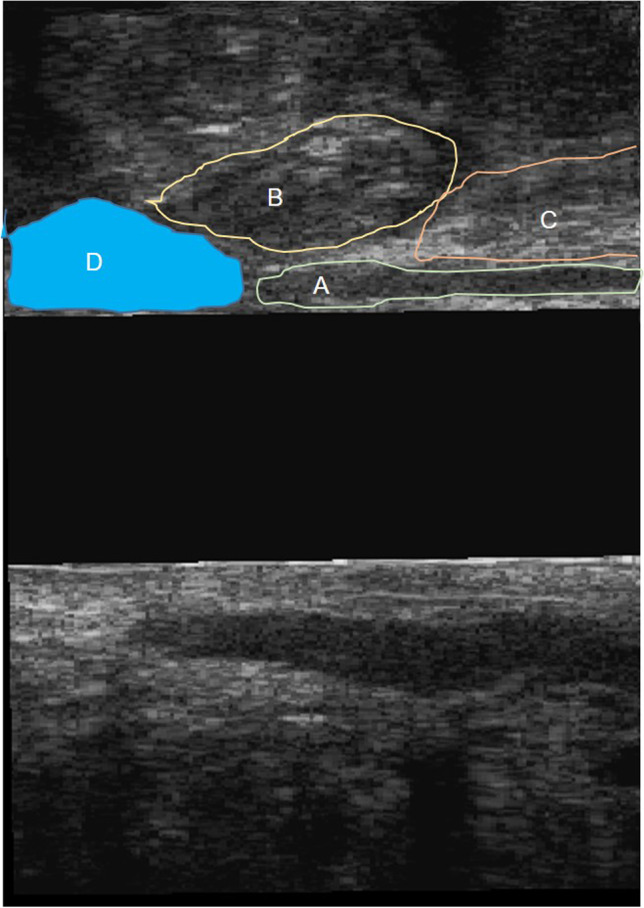
Endoanal ultrasound image showing a sagittal section of an anterior post-fistulotomy external and internal anal sphincter defects and remaining sphincters. **A** internal anal sphincter, **B** external anal sphincter, **C** puborectalis muscle, **D** fistulotomy defect

All 3D-EAUS were performed by the same surgeon during a second visit to the outpatient clinic. The B&K Medical Systems Pro Focus 2202® and B-K 2050 transducer (B-K Medical, Herlev, Denmark) were used. A preoperative diagnostic ultrasound and 8-week post-fistulotomy scan was performed for all patients. Patients were examined in the prone jack-knife position, and the scan was systematically performed from the upper to the lower thirds of the anal canal. An initial 2D-EAUS was carried out and immediately followed by a 3D-EAUS examination. All examinations were performed at a 10 MHz frequency with 0.2 mm spacing taking 300 sequential images which were subsequently reconstructed in a 3D cube. These examinations were repeated after instillation of 10% hydrogen peroxide in patients who had an open EO. Quantitative measurements of the following variables were taken in all patients: total length of the anal canal, total length of the EAS and internal anal sphincter (IAS), preoperative sphincter involvement (Fig. 1) and postoperative sphincter defect for both the EAS and IAS (Fig. 2). The percentage of sphincter length involved or divided pre- and postoperatively was calculated for both sphincters. Whilst the 3D-EAUS is performed, it is important to keep the patient’s buttocks separated in order to obtain accurate images of the inferior third of the anal canal which could otherwise be confused with subcutaneous tissue, thus overestimating the total length of the anal canal and EAS. The separation between the EAS and puborectalis muscle can be seen on midline sagittal section as a thin hypoechoic line, which combined with the transverse axial image, defines the exact length of the EAS. When the fistula did not cross perpendicular to the EAS, a midway measure to calculate the length of EAS involved was taken. The proximal limit of the IAS was the anorectal junction.

### Surgery

All patients included in the study were operated on in the prone jack-knife position. Patients were prepped with an enema and locoregional anaesthesia was used. 3D-EAUS was used to guide surgery, but final decision to perform a fistulotomy was the surgeon’s criteria. An initial physical examination was performed followed by fistulotomy taking note of IO and fistula location and height.

### Quality of life assessment

Patients completed QOL (SF-36 [11] and Faecal Incontinence Quality of Life Score FIQOLS [12]) pre, immediately postoperatively, and 6 and 12 months after surgery.

### Statistical analysis

Spearman’s correlation coefficient was used to correlate the preoperative fistula height with the extent of fistulotomy. The Wilcoxon test was used to compare pre and postoperative continence. The chi-squared test or Likelihood ratio were used to assess the level of division of the sphincters and deterioration in anal continence. A *p* value greater than 0.05 was considered statistically significant. Statistical analysis was performed using the IBM® SPSS® version 26 for Windows (SPSS, Chicago, Illinois, USA).

## Results

**Fig. 3:**
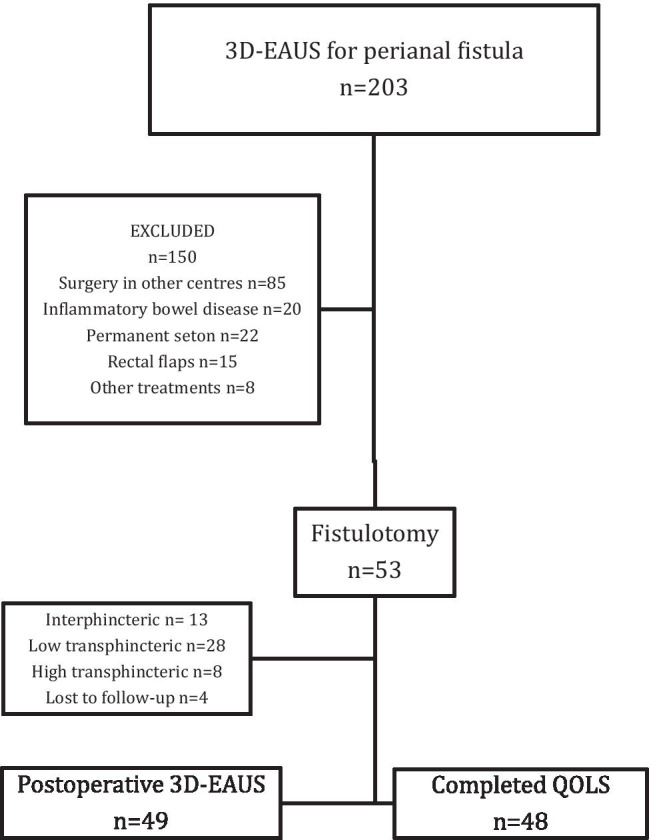
Patient flow diagram. 3D-EAUS = three dimensional endoanal ultrasonography; QOLS = quality of life scales

**Fig. 4:**
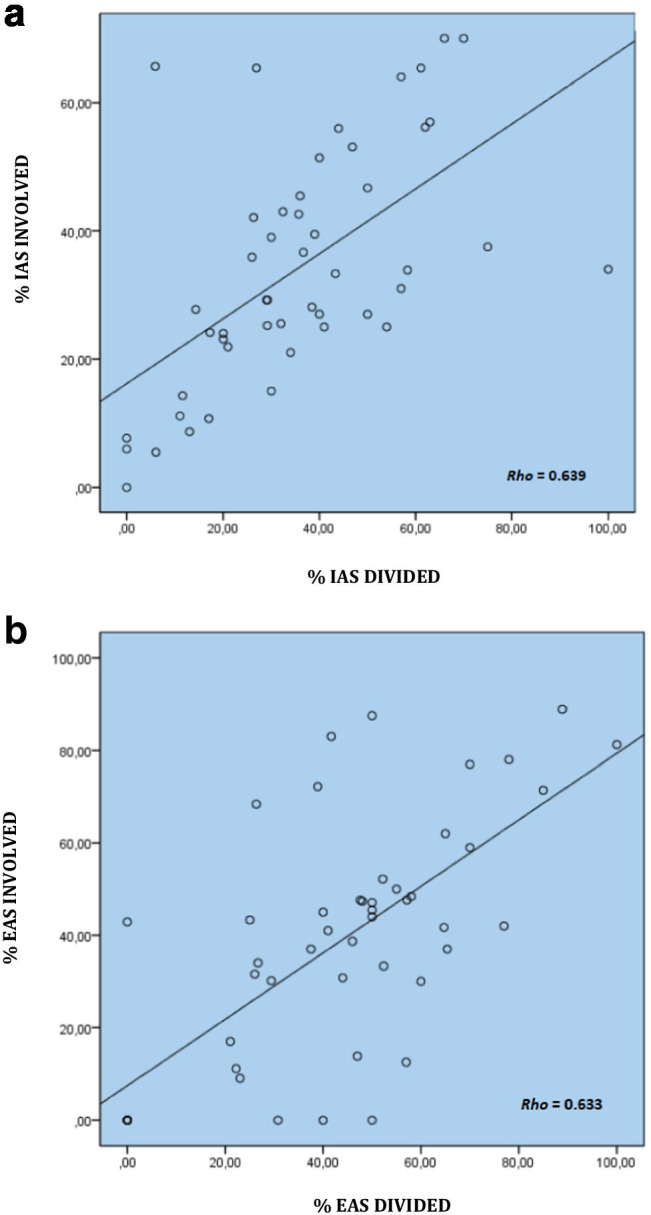
Three-dimensional endoanal ultrasound measurements and their correlation between the percentage of involved sphincter and percentage of divided sphincter pre- and postoperative respectively. **a** Internal anal sphincter. **b** External anal sphincter. (Spearman’s correlation *p* < 0.001)

Patient distribution is shown in Fig. 3. A total of 49 patients (37 male and 12 female) with a mean age of 49 years (range 21–77) were selected. Patient characteristics are listed in Table 1. Thirteen patients had intersphincteric tracts, 28 low transsphincteric tracts and 8 high transsphincteric tracts in this group of patients. Preoperative 3D-EAUS measurements and the percentage of IAS and EAS involved pre- and postoperatively are shown in Table 2. There was a strong correlation between preoperative sphincter involvement and postoperative sphincter division, with no significant differences between pre- and postoperative values (Spearman’s correlation *IAS Rho* = 0.639; *EAS Rho* = 0.633, *p* < 0.001) (Fig. 4a and b).

**Table 1:** Patient characteristics

Total		*n* = 49
Mean length of symptoms (months) (range) *	11.9 (0–120)	
Drained abscesses	25 (51)	
Loose seton	13 (26.5)	
Fistulotomy	3 (6.1)	
Fistulectomy	1 (2)	
Internal lateral sphincterotomy	5 (10.2)	
Hemorrhoidectomy	3 (6.1)	
Female		*n* = 12
Vaginal deliveries	6 (0)	
Episiotomy	3 (25)	
Hysterectomies	1 (8,3)	

Values are expressed as *n* (%) unless otherwise staed *from diagnosis to surgery

**Table 2:** Pre- and postoperative three-dimensional endoanal ultrasound measurements of sphincter involvement and division, respectively

	IAS		EAS	
	Median % (range)	Median mm (range)	Median % (range)	Median mm. (range)
Preoperative	31 (0–66)	9 (0–24)	41.2 (0–89)	10 (0–28)
Postoperative	32,4 (0–75)	8 (0–25)	41.7 (0–100)	11 (0–29)

*IAS* internal anal sphincter; *EAS* external anal sphincter *p* > 0.05

**Fig. 5:**
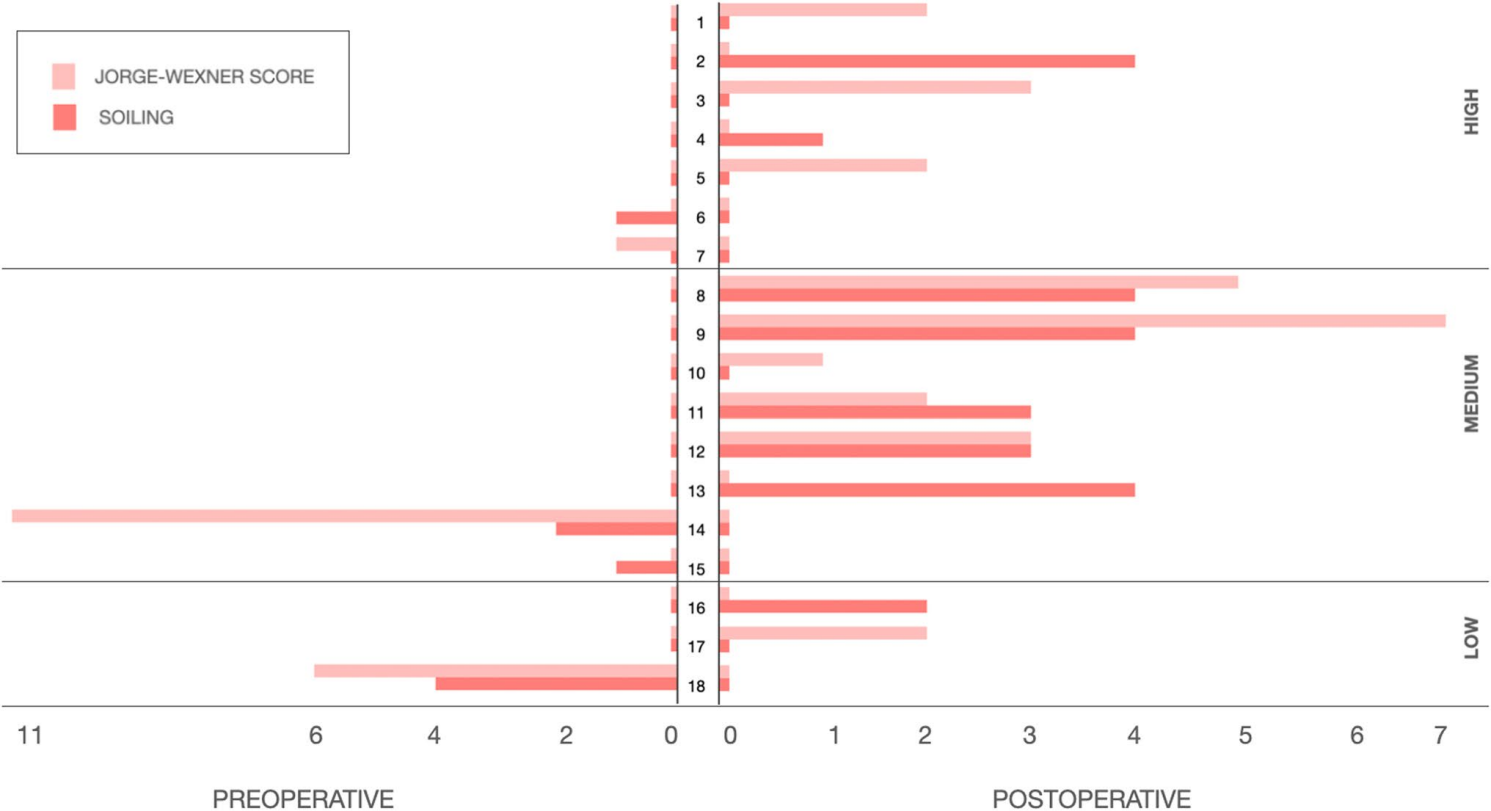
The relationship between the amount of sphincter involved and the Jorge-Wexner score prior to and 1 year after surgery

Anal continence was analysed in relation to the percentage of sphincter divided during fistulotomy. There were significant differences with regards to the thirds of the EAS divided (Likehood ratio = 0.049). When division of the EAS was less than 66%, only 8/33 patients (24.2%) showed deterioration in FI (including mild incontinence) or soiling. However, when more than 66% of the anal sphincter was divided, 5/8 patients (62.8%) showed deterioration in FI (Tables 3 and 4). Division of over 50% of the IAS was associated with significant differences in FI pre- and postoperatively (Table 5). Five of the 8 cases with a deterioration in FI had a division of over 50% and 66% of the IAS and EAS, respectively. Figure 5 shows the relationship between the amount of sphincter involved and the Jorge-Wexner score prior to and 1 year after surgery.

**Table 3:** Subdivision of the level of division of the external anal sphincter and immediate postoperative deterioration in anal continence defined as any extent of impairment of faecal incontinence. Divided into thirds

		Deterioration in anal continence	
		No	Yes
EAS	Low	11(30.6)	2 (15.4)
	Medium	22 (61.1)	6 (46.2)
	High	3 (8.3)	5 (38.5)
Total		36	13

Values are expressed as *n* (% of subgroup deterioration in anal continence)

low (≤ 33%); medium (33–66%); high (≥ 66%). *EAS* external anal sphincter. *p* = 0.049

**Table 4:** Subdivision of the level of division of the external anal sphincter and immediate postoperative deterioration in anal continence defined as any extent of impairment of fecal incontinence. Divided into subgroups (below or above 66%)

		Deterioration in anal continence	
		No	Yes
EAS	< 66%	33 (91.7)	8 (61.5)
	≥ 66%	3 (8.3)	5 (38.5)
Total		36	13

Values are expressed as *n* (% of subgroup deterioration in anal continence)

EAS external anal sphincter. *p* = 0.018

**Table 5:** Subdivision of the level of division of the internal anal sphincter (below or above 50%) and deterioration in anal continence defined as any extent of impairment of faecal incontinence in the immediate postoperative period

		Deterioration in anal continence	
		No	Yes
IAS	< 50%	32 (88.9)	5 (38.5)
	≥ 50%	4 (11.1)	8 (61.5)
Total		36	13

Values are expressed as *n* (% of subgroup deterioration in anal continence)

*IAS* internal anal sphincter, *p* = 0.001

Five (10.2%) patients had some degree of preoperative FI, and 8 (16.3%) had a deterioration in Jorge-Wexner score 1 year after surgery, of which 6 had mild incontinence with a Jorge-Wexner score < 3. The 2 patients with a major degree of incontinence were the following: the first case had a transsphincteric fistula which crossed above 47.4% of the EAS and 26.9% of the IAS pre, with division of 70% of the EAS and 50% of the IAS postoperatively. The second case had a fistula that involved 47.6% of EAS and 29% of the IAS pre with division of 48% of the EAS and 29.2% of the IAS divided postoperatively. No patients had a Jorge-Wexner score > 4 at 1-year follow-up.

**Fig. 6:**
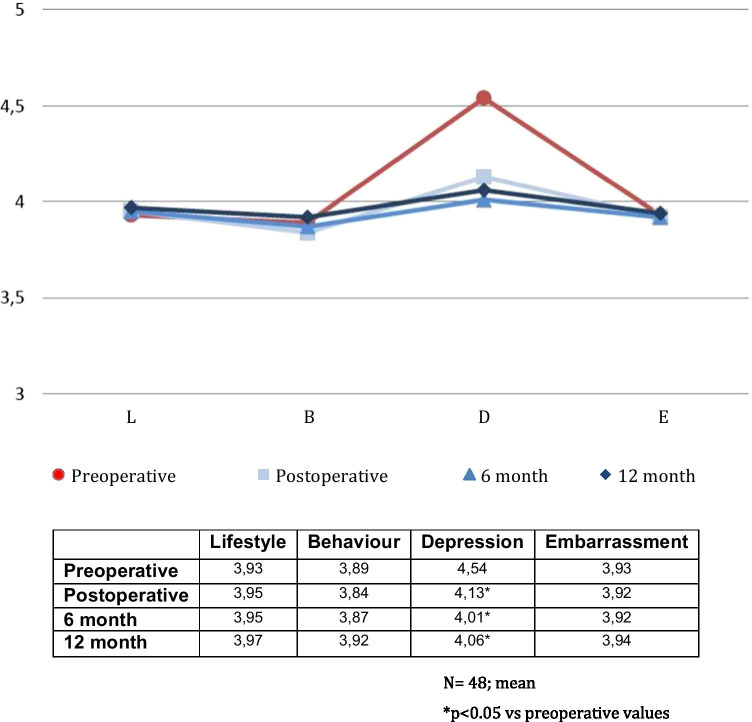
Preoperative, immediate postoperative and 6- and 12-month faecal incontinence quality of life core (L, lifestyle; B, behaviour; D, depression; E, embarrassment)

**Fig. 7:**
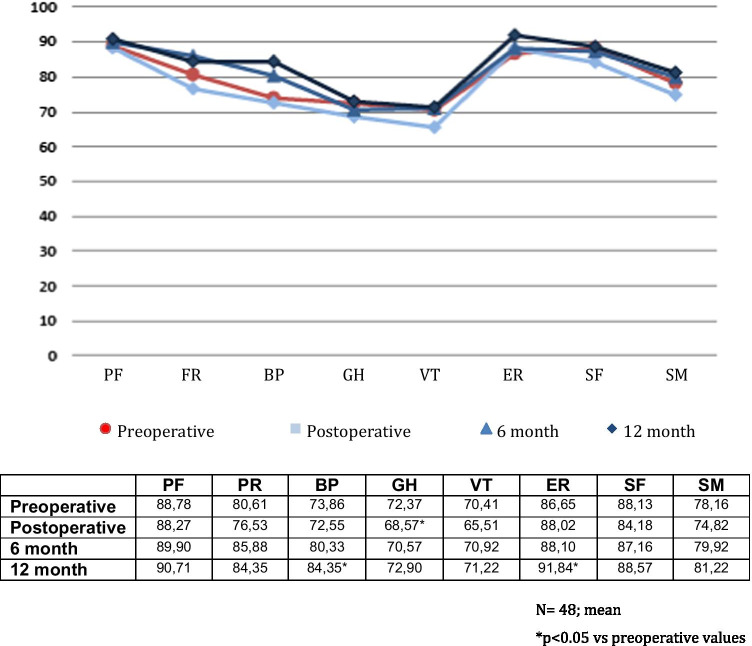
Preoperative, immediate postoperative and 6- and 12-month SF-36 score (PF, physical function; RF, physical role; BP, body pain; GH, general heath; VT, vitality; ER, emotional role; SF, social function; SM, mental health)

One patient did not complete the QOL questionnaires. QOL analysis of 48 patients showed no significant differences in FIQOL in the immediate, 6- and 12-month follow-ups (*p* > 0.05). Figure 6 shows the results of the FIQOLS. All domains showed an initial deterioration which slowly improved over time. Depression was the only domain which showed a statistically significant deterioration and improvement. The SF-36 score showed an improvement at annual follow-up, most evident in the body pain and emotional role areas, both of which were statistically significant as shown in Fig. 7. There was 1 fistula recurrence.

## Discussion

The present study shows a strong correlation between pre- and postoperative, IAS and EAS measurements. A median length of 41% of EAS and 32% of IAS was divided during fistulotomy without a significant long-term deterioration in FI, soiling or QOLS. Division of the lower two-thirds of the EAS was associated with a deterioration in FI in 8/33 (24,2%) of patients, whilst division above this cut-off point was associated with a deterioration in FI in 5/8 (62,8%) of patients. There are currently no studies using 3D-EAUS to quantify the extent of sphincter division after fistulotomy and correlate this with postoperative FI. A study in 40 patients which evaluates fistulotomies/fistulectomies with 2D-EAUS concludes that fistulotomies produce less damage to the sphincters, but there is no mention of cure or incontinence rates [13]. Voyvodic et al. published a study in 330 patients and found no correlation between the extent of fistulotomy measured by 2D-EAUS, manometry and postoperative incontinence [14]. These studies were performed using 2D-EAUS, a subjective, observer-dependant technique which fails to objectively quantify sphincter division, as opposed to 3D-EAUS. Recently, Murad-Regadas published the results of a cohort of patients with less than 40–50% of EAS involvement preoperatively, who were scheduled for fistulotomy, and reported a 33% rate of FI but did not correlate the length of division with FI [4]. The present study reports the value of 3D-EAUS to quantify the length of IAS and EAS divided during fistulotomy and its correlation with postoperative FI. These measurements are helpful to indicate the appropriate surgical technique for each type of fistula, minimizing the risk of postoperative FI and recurrence. The same group published a study in 2012 conducted in 36 fistulotomy patients and found a strong correlation between preoperative 3D-EAUS measurement of fistula height with intra and postoperative 3D-EAUS measurement of IAS and EAS division [5]. Similar results were found in 16 patients undergoing rectal mucosal advancement flap for perianal fistulae [15]. 3D-EAUS therefore becomes a fundamental tool for pre- and postoperative evaluation of perianal fistulae and sphincter division.

Some authors have modified the Parks classification dividing fistulas in intersphincteric, low, mid and high transsphincteric, suprasphincteric and extrasphincteric [16]. The subdivision of transsphincteric fistulas according to the level at which they cross the EAS, dividing the latter in thirds tends to be arbitrary. Like other authors, we believe fistulotomy is the treatment of choice for intersphincteric and low transsphincteric fistulas, but those that affect the mid or high portions of the EAS remain a surgical challenge [17].

FI was mild for all patients with a Jorge-Wexner score < 4 in all cases, similar to the FI rates ranging from 7 to 44% reported by other authors [2, 4, 18–20]. The aetiology of FI is multifactorial, and the rate of reported incontinence also depends on the definition and arduousness with which it is sought. We took into account even the mildest forms of incontinence. In addition, there was no significant difference between pre- and postoperative FIQOLS in the long term except for depression which showed a significant deterioration and subsequent improvement. Likewise, all domains of the SF-36 scores deteriorated in the immediate postoperative period and improved to baseline or above values 1 year after follow-up. Significant differences in the emotional role, general health or body pain scores could be explained by the fear of surgery-related incontinence or that patients were more aware of their body in the immediate postoperative period just after going through surgery. Other authors have found that the severity of incontinence increases with the complexity of the fistula, negatively influencing quality of life in 141 patients who underwent fistula surgery [21].

2D-EAUS has also been used to evaluate the extent of sphincterotomy for treatment of chronic anal fissure and compare this to postoperative incontinence and other clinical parameters [22–25]. 3D-EAUS allows us to quantitatively measure the length of lateral internal sphincterotomy or fistulotomy, determining the percentage of sphincter which has been divided and correlate this with postoperative results. 3D-EAUS therefore becomes a fundamental tool for pre and postoperative evaluation of perianal fistulae and sphincter division.

This study has several limitations such as the number of patients included even though this is equal to or superior to other studies [26, 27]. All scans and measurements in this study have been performed by the same surgeon, which can reduce interobserver variability on the one hand but also be a source of bias. Notwithstanding, the strengths of this study include the thorough pre- and postoperative evaluation of fistulotomies with 3D-EAUS and extensive search for and follow-up of postoperative FI using the Jorge-Wexner score and QOLS.

In conclusion, 3D-EAUS is a valuable tool for quantifying the extent of sphincter involvement pre- and postoperatively. Post-fistulotomy faecal incontinence is mild and increases with increasing length of sphincter division, with the highest probability when over two-thirds of the EAS is divided. This does not affect long-term quality of life.

## Data Availability

Data is available on request.
